# *Toxoplasma gondii* Infection in Seagull Chicks Is Related to the Consumption of Freshwater Food Resources

**DOI:** 10.1371/journal.pone.0150249

**Published:** 2016-03-14

**Authors:** Oscar Cabezón, Marta Cerdà-Cuéllar, Virginia Morera, Ignacio García-Bocanegra, Jacob González-Solís, Sebastian Napp, Maria P. Ribas, Berta Blanch-Lázaro, Xavier Fernández-Aguilar, Noelia Antilles, Sergio López-Soria, Cristina Lorca-Oró, Jitender P. Dubey, Sonia Almería

**Affiliations:** 1 Servei d'Ecopatologia de Fauna Salvatge, Departament de Medicina i Cirurgia Animals, Facultat de Veterinaria, Universitat Autònoma de Barcelona, Bellaterra, Spain; 2 Centre de Recerca en Sanitat Animal (CReSA) - Institut de Recerca i Tecnologia Agroalimentàries (IRTA), Campus de la Universitat Autònoma de Barcelona, Bellaterra, Barcelona, Spain; 3 Institut de Recerca de la Biodiversitat (IRBio) and Departament de Biologia Animal, Universitat de Barcelona, Barcelona, Spain; 4 Departamento de Sanidad Animal, Facultad de Veterinaria, UCO, Córdoba, Spain; 5 Animal Parasitic Diseases Laboratory, Beltsville Agricultural Research Center, Agriculture Research Service, United States Department of Agriculture, Beltsville, MD, United States of America; 6 Departament de Sanitat i d’Anatomia Animals, Facultat de Veterinària, Universitat Autònoma de Barcelona, Bellaterra, Spain; CEFE, FRANCE

## Abstract

Understanding the spread of *Toxoplasma gondii* (*T*. *gondii*) in wild birds, particularly in those with opportunistic feeding behavior, is of interest for elucidating the epidemiological involvement of these birds in the maintenance and dissemination of the parasite. Overall, from 2009 to 2011, we collected sera from 525 seagull chicks (Yellow-legged gull (*Larus michahellis*) and Audouin’s gull (*L*. *audouinii*)) from 6 breeding colonies in Spain and tested them using the modified agglutination test (MAT) for the presence of antibodies against *T*. *gondii*. Chick age was estimated from bill length. Main food source of seagull chicks was evaluated using stable isotope analyses from growing scapular feathers. Overall *T*. *gondii* seroprevalence was 21.0% (IC_95%_ 17.5–24.4). A generalized linear mixed-effects model indicated that year (2009) and food source (freshwater) were risk factors associated to the individual risk of infection by T. gondii, while age (days) was close to significance. Freshwater food origin was related to the highest seroprevalence levels, followed by marine origin, supporting freshwater and sewages as important routes of dispersion of *T*. *gondii*. Year differences could indicate fluctuating rates of exposure of seagull chicks to *T*. *gondii*. Age ranged from 4 to 30 days and seropositivity tended to increase with age (P = 0.07), supporting that seropositivity is related to T. gondii infection rather than to maternal transfer of antibodies, which in gulls is known to sharply decrease with chick age. This study is the first to report *T*. *gondii* antibodies in Yellow-legged and Audouin’s gulls, thereby extending the range of intermediate hosts for this parasite and underscoring the complexity of its epidemiology.

## Introduction

*Toxoplasma gondii* is a zoonotic intracellular protozoan parasite of worldwide distribution [1]. Wild and domestic felids are the definitive hosts and therefore are the only known hosts that excrete oocysts in feces. Humans and virtually all warm-blooded species, including birds, can be intermediate hosts and become infected by the ingestion of food and water contaminated with sporulated *T*. *gondii* oocysts, by consumption of tissue cysts in infected animal tissues, or congenitally [1]. *T*. *gondii* has been also recently considered as a waterborne parasite, and has been detected in diverse water sources including those used as recreational and drinking for humans [2]. Contamination of near-shore waters by *T*. *gondii* oocysts has been proven to be a threat for several marine mammal species, such as the sea otters (*Enhydra lutris*) from the California coasts, USA [3]. Sewage is considered to be one of the main sources of *T*. *gondii* oocysts from land to coastal environments [4]. However, the role of freshwater runoffs and/or sewages in the epidemiology of *T*. *gondii* in the Iberian Peninsula, as well as its impact in the health status on the different warm blooded species linked to aquatic ecosystems, have not been yet evaluated.

Birds can be exposed to the parasite via ingestion of food or water contaminated with sporulated oocysts and with infected tissues, based on their feeding habits. Although it is not common, vertical infection by *T*. *gondii* has been reported in some bird species [5,6]. Indeed, *T*. *gondii* infection is prevalent in many domestic and wild avian species, although the epidemiological role of those species is poorly understood [1,5,7,8,9]. Birds are suspected to act as dispersive agents of *T*. *gondii* into isolated territories without felines [10]. For instance, seropositivity to *T*. *gondii* has been reported in Arctic fox (*Alopex lagopus*) and polar bear (*Ursus maritimus*) from the Svalbard Island (Norway) [10,11,12,13], apparently in the absence of felids. However, the effectiveness of wild birds as carriers of the parasite into isolated feline-free environments is not fully understood.

Among birds, scavenging species such as some seagulls, which regularly feed on refuse dumps and sewage water, would be good candidates to study their epidemiological importance in *Toxoplasma* infection, but have rarely been studied. Exploring the spread of *T*. *gondii* in wild birds and possible links with opportunistic feeding behavior can help understanding the role of birds in maintaining and disseminating parasites over large geographical areas [5,6]. The feeding habits and the ecological adaptability to anthropized habitats of *Larus* spp. make them suitable to be considered as sentinels for environmental public health risks.

In the present study, we assayed *T*. *gondii* antibodies in seagull chicks from several breeding colonies in Spain. Main aims of the study were (1) to assess the role of seagulls as intermediate hosts and reservoirs of *T*. *gondii*; (2) to explore factors related to the presence of antibodies against this protozoa parasite in seagulls, particularly in relation to their feeding habits and access to human waste.

## Materials and Methods

### Animals and Samples

From 2009 to 2011, 479 yellow-legged gull chicks (*Larus michahellis*) were sampled from the Ebro Delta (n = 102), three Mediterranean islands (Medes Islands, n = 96; Dragonera Island, n = 68; and Columbretes Islands, n = 143) and one Atlantic island (Ons Island, n = 70) of the Iberian Peninsula (Table 1; Supplementary data). In 2011, we also sampled 47 Audouin’s gull (*Larus audouinii*) chicks from Alboran Island (Western Mediterranean). Colonies were sampled in a single visit over 1 to 4 days. All colonies are close to inhabited territories with the exception of Alboran Island and Columbretes Island, both small islands located more than 50 km away from continental land. All breeding colonies, except the one at Ebro Delta, are located in feline-free islands, with no wild or domestic felids or watercourses.

**Table 1:** Seroprevalence of *Toxoplasma gondii* in seagulls from Spain.

Species	Location (island)	UTM	Year	Age (Mean±SD)	Main food source	No. examined/positive (%
Yellow-legged Gull (*Larus michahellis*)	Columbretes	42°03′00″N / 03°13′15″E	2009	20.7 ± 4.3	Marine	86/39 (45.3)
			2010	20.1 ± 3.8	Marine	32/3 (9.4)
			2011	16.3 ± 3.8	Marine	25/3 (12.0)
	Delta	39°35'13.49"N / 2°19'19.71"E	2009	20.9 ± 3.7	Marine	32/10 (31.3)
			2010	13.7 ± 4.2	Marine	32/10 (31.3)
			2011	15.2 ± 3.3	Marine	38/0 (0.0)
	Dragonera	39°53′53″N / 0°41′07″E	2010	11.1 ± 4.3	Marine	32/1 (3.1)
			2011	15.2 ± 3.2	Freshwater	36/4 (11.1)
	Medes	42°22'28.31"N / 8°56'7.69"O	2009	21.5 ± 3.2	Freshwater	32/16 (50.0)
			2010	20.9 ± 3.4	Freshwater	32/5 (15.6)
			2011	16.3 ± 3.1	Freshwater	32/14 (43.8)
	Ons	40°43′12″N / 0°51′47″E	2010	14.2 ± 4.2	Marine	32/1 (3.1)
			2011	13.8 ± 6.7	Marine	38/3 (7.9)
Audouin’s Gull (*Larus audouinii*)	Alboran	35°56'20.51"N / 3° 2'7.79"O	2011	NA^a^	Marine	46/1 (2.2)

^a^ NA: not available.

A single fledgling from each brood was captured, blood sampled, measured, weighed and marked with paint. The animals were sampled in the field and released immediately after sampling. Since maternal antibody concentration decreases with age in seabird nestlings, including gulls [14,15], we estimated the age of each chick from bill length. In yellow-legged gulls bill growth is known to approach linearity from the first to the fifth week following the relationship: age (days) = bill length (mm)*0.963–22.34 (JGS unpublished data), resulting in a chick age ranging from 4 to 30 days. Blood was extracted from a tarsal or brachial vein using sterile syringes, collected in a 2 ml tubes without anticoagulant and maintained in a cooler while in the field. In the lab, the blood was centrifuged at 652 g for 15 min and the resulting sera were stored at -80°C until analysis. Six to eight breast feathers from each bird were also collected and stored in plastic bags until analyzed.

Sampling methods were in compliance with the Ethical Principles in Animal Research of the *Universitat Autònoma de Barcelona* and field permits were authorized by *Generalitat de Catalunya*, *Govern de les Illes Balears*, *Generalitat Valenciana*, *Xunta de Galicia and Junta de Andalucia*. All sampling procedures and/or experimental manipulations in the field were reviewed and approved as part of obtaining the field permit.

### Stable Isotopes Analyses

Before analyses, feathers were cleaned washing them in a 0.25M sodium hydroxide solution, and all residues eliminated by rinsing them repeatedly with distilled water. Once cleaned, feathers were dried to constant mass in an oven at 46°C and grounded to powder in a freezer mill (Spex Certiprep 6750; Spex Industries Inc., Metuchen, New Jersey, USA), operating at liquid nitrogen temperature. The resulting feather powder was subsampled for Carbon and Nitrogen analyses (0.4 mg) and for Sulphur analyses (3.4 g), placed in tin capsules and crimped for combustion. The samples were oxidized in a Flash EA1112 (for C and N) and EA1108 (for S) coupled to a Delta C stable isotope mass spectrometer through a Conflo III interface (Thermo Finnigan, Bremen, Germany), which was used to determine the δ^15^N, δ^13^C and δ^34^S values. Isotope ratios are expressed in δ notation as per mil units (‰) according to the following equation (δX = [(Rsample/Rstandard)-1] x 1000) where X (‰) is ^15^N, ^13^C or ^34^S and Rsample is the corresponding ratio 15N/14N, 13C/12C or 34S /32S. We used atmospheric nitrogen (AIR), Vienna-Pee Dee Belemnite (V-PDB) and troilite from the Canyon Diablo Meteorite, as Rstandard for the ^15^N, ^13^C and ^34^S, respectively. International standards were inserted every 12 samples to calibrate the system and compensate for drift over time. Sample errors were estimated by replicating essays of the standard material, indicating a sample error of ± 0.1‰ for the C and ± 0.2‰ for the N and S, although these values might be higher for organic materials of high complexity, such as feathers. All isotopic analyses were performed at the isotopic ratio mass spectrometry facility at the “Serveis Científico-Tècnics” at Barcelona’s University.

Isotopic values of the food sources were obtained from spontaneous regurgitations from chicks and fish discarded from vessels, collected by Ramos et al. [16] in a previous study of the Mediterranean colonies and by Moreno et al. [17] for the Atlantic colony, and classified in four functional groups: marine, freshwater and terrestrial prey and dumpsite feeding for the Mediterranean colonies and three groups (marine and terrestrial prey and dumpsite feeding) for the Atlantic colony, as freshwater prey was not found in those regurgitates. Trophic enrichment values were also taken from published data following Ramos et al. [18].

To assess the contribution of each food group to the chicks’ diet a three-isotope Bayesian mixing model was used (SIAR, Stable Isotope Analysis in R [19]) in the R Environment (R Core Team, 2014). This model includes the standard deviation of the isotopic values as a variable, thus allowing the intracolony variability to be taken into account, and applies a Markov Chain Monte Carlo (MCMC) method with a Dirichlet distribution. We ran the model with 10^6^ iterations, discarding the first 10^5^ as a burn-in.

### Serological test

Sera were examined by the modified agglutination test (MAT) to detect IgG antibodies, which in birds would be mainly IgY, against *T*. *gondii*. Sera were tested at 1:25, 1:50, 1:100 and 1:500 dilutions. Positive and negative controls were also included in all tests. Titres of 1:25 or higher were considered positive and those with doubtful results were re-examined. This technique has been previously evaluated in several bird species [5,20,21].

### Definition of variables and Statistical analysis

Prevalence of antibodies against *T*. *gondii* was estimated from the ratio of positive to the total number of samples, with the exact binomial confidence intervals of 95% [22]. Due to the limited number of Audouin’s gulls sampled (n = 47), the associated risk factors could not be properly analyzed, so this analysis was restricted to yellow-legged gulls, the most abundant and widely distributed gull species in Spain. To test for the potential influence of seroprevalence on chick body condition we checked for normality and equality of variances and performed an ANCOVA analysis, with body mass as dependent variable, and sampling (year/locality) and seroprevalence status as factors. To obtain an indication of the relevance of sampling year, food source and age on the risk of a chick being seropositive, we first tested their association to seropositivity for *T*. *gondii* using chi-square (sampling year, food source) and ANOVA tests (age). Associations between *T*. *gondii* seroprevalence and the three explanatory variables were analyzed using a Generalized Linear Mixed Model (GLMM) with an underlying binomial distribution (log link). The colony was included as a random effect. Models were fitted by Laplace approximation, implemented in the glmer function of the lme4 package for R (http://CRAN.R-project.org/package=lme4) [23]. Inference was based on model comparison of nested models (ANOVA), and the process of model selection was based on the lowest Akaike information criterion (AIC) value. Statistical analyses were carried out in R software (http://www.r-project.org/).

## Results

Overall seroprevalence (MAT ≥ 1:25) against *T*. *gondii* was 21.0% (CI_95%_: 17.5–24.0), with titres of 1:25 in 38.0% of the samples, 1:50 in 39.4% of the samples, 1:100 in 18.3% of the samples and 1:500 in 4.2% of the samples. The seroprevalence in yellow-legged chicks and in Audouin’s gull chicks were 22.8% of 479 chicks and 2.2% of 46 chicks, respectively (Table 1).

Mean age of yellow-legged gull chicks differed significantly among the 13 samplings (locality-year) (Table 1). Body mass differed significantly among colonies and with chick age, but did not differ between seropositive and seronegative chicks (ANCOVA, locality-year F_12,452_ = 5.79, *P*<0.001; age F_1,452_ = 674.37, *P*<0.001; seroprevalence status F_1,452_ = 0.44; P = 0.51; locality-year*infection status F_11,452_ = 0.59; *P* = 0.84).

Statistically significant differences between the seropositivity to *T*. *gondii* and the three explanatory variables (year, food source and age) were observed in the bivariate analysis. Significantly higher seropositivity was found in 2009 (43.3%; 65/150) compared to 2010 (12.5%; 20/160) and 2011 (14.2%; 24/169). Freshwater origin as main food source showed statistically higher risk of infection compared to marine origin (29.5% of 132 seagull chicks versus 20.2% of 347 seagull chicks, respectively). The ANOVA tests showed that seropositivity significantly increased with age (p <0.001).

The model with best AIC-value (463.6) indicated that year (2009) and food source (freshwater) were risk factors associated to the individual risk of infection by *T*. *gondii*, while age (days) was close to significance (Table 2).

**Table 2:** Results of the generalized linear mixed-effects model of risk factors associated to *Toxoplasma gondii* seroprevalence in seagulls in Spain.

Variable	β (*S*.*E*.)	*p*-value	df	OR (CI_95%_)
Food source				
Marine prey	a	a		a
Freshwater prey	0.69 (0.26)	0.009*	1	1.99 (1.02–4.14)
Year				
2009	1.42 (0.33)	2.2e-05*	1	4.14 (2.08–8.11)
2010	-0.07 (0.34)	0.82	1	0.93 (0.47–1.80)
2011	a	a		a
Age (days)	0.05 (0.33)	0.075	1	1.05 (0.99–1.11)

^a^ Reference category. df: degree freedom

* Statistically significant

Food resources according to their origin (marine, freshwater, terrestrial, refuse) fed by chicks from the different seagull colonies is indicated in Table 3.

**Table 3:** Diet reconstruction with Bayesian mixing model: percentage of use of main food sources for each breeding colony and year. Given values are medians, with 95% credibility intervals in brackets. Values in bold represent the main food source; underlined values represent the secondary source.

Location (island)	Year	Freshwater	Marine	Refuse	Terrestrial
Alboran	2009	0.194 (0.04–0.31)	**0.716 (0.63–0.79)**	0.03 (0–0.11)	0.053 (0–0.15)
Columbretes	2009	0.291 (0.14–0.42)	**0.603 (0.51–0.7)**	0.064 (0–0.16)	0.032 (0–0.1)
	2010	0.227 (0.03–0.43)	**0.634 (0.44–0.79)**	0.088 (0–0.26)	0.034 (0–0.14)
	2011	0.249 (0.08–0.41)	**0.626 (0.5–0.74)**	0.081 (0–0.2)	0.034 (0–0.11)
Delta	2009	0.271 (0.07–0.46)	**0.581 (0.42–0.72)**	0.089 (0–0.24)	0.045 (0–0.15)
	2010	0.317 (0.1–0.5)	**0.513 (0.3–0.65)**	0.13 (0.01–0.28)	0.029 (0–0.12)
	2011	0.292 (0.09–0.47)	**0.558 (0.41–0.7)**	0.092 (0.02–0.34)	0.045 (0–0.15)
Dragonera	2010	0.215 (0.03–0.43)	**0.574 (0.42–0.1)**	0.167 (0.02–0.34)	0.031 (0–0.13)
	2011	**0.491 (0.25–0.75)**	0.154 (0.02–0.31)	0.286 (0.15–0.41)	0.053 (0–0.18)
Medes	2009	**0.48 (0.25–0.7)**	0.304 (0.18–0.43)	0.136 (0.02–0.29)	0.068 (0–0.2)
	2010	**0.428 (0.19–0.72)**	0.157 (0.02–0.33)	0.292 (0.13–0.43)	0.101 (0.01–0.28)
	2011	**0.655 (0.45–0.81)**	0.079 (0.01–0.19)	0.221 (0.13–0.32)	0.04 (0–0.19)
Ons	2010	NA	**0.532 (0.43–0.62)**	0.44 (0.32–0.55)	0.022 (0–0.11)
	2011	NA	**0.756 (0.66–0.88)**	0.143 (0.02–0.27)	0.086 (0–0.25)

## Discussion

The present study shows the first data on seroprevalence of *T*. *gondii* in yellow-legged and Audouin’s gulls worldwide, and adds two new species of gulls to the list of possible intermediate host for *T*. *gondii* infection. Isolation of the parasite from tissues would be necessary to confirm the intermediate host role of these seagull species. Of the two species of seagull analyzed, Audouin’s gull is an endemic species from the Mediterranean, which is regarded as globally near threatened [24]. The *T*. *gondii* seroprevalence differences between the two seagull species seem not to be due to differences in feeding behavior since similar origin of food was observed in the colony from Audouin´s gull compared to the yellow-legged colonies in the Mediterranean areas (Table 1). These differences could be due to the fact that Audouin´s gull, which showed the lowest seroprevalence in the study, were sampled in Alboran Island, a small Mediterranean island located far from the continental land, and closer to the Northern African (64.82 Km) than to the Spanish (92.6 Km) coasts. However, since the two species were sampled in different colonies, it is not possible to confirm this hypothesis.

Seroprevalence of *T*. *gondii* antibodies in this study showed also statistically significant differences among sampling years. The highest seroprevalence in yellow legged seagull chicks was observed from samples collected during the first year of study compared to those from the next two years, although different dynamics were observed in some colonies among years. These results would indicate fluctuating rates of transmission of *T*. *gondii* as has been observed in other wildlife species in Spain [25]. The highest seroprevalence observed in 2009 was not related to a particular colony, since no significant differences in seroprevalence were observed among colonies that year, and the variations seemed to be independent of the origin of the diet. Temporal, year-to-year variations have also been explained by the effect of variable meteorological conditions on the level of infection in domestic [26] and wild species [27]. Although seroprevalence fluctuation in wildlife populations has been studied [28], further studies will be needed to explain seroprevalence of *T*. *gondii* antibodies fluctuations in wildlife species.

The presence of antibodies against *T*. *gondii* in seagull chicks of the colonies from unoccupied and feline-free islands can be explained by 1) the presence of maternal antibodies or 2) by horizontal infection of chicks while being fed by their progenitors. Even though in the present study seropositivity seemed to increase with age, which would support the second hypothesis, the association was not statistically significant at the 5% level. However, the p value was close to significance (p value of 0.075) and the best fitting model (lowest AIC) included age, indicating that this variable is relevant for the evaluation of the risk *T*. *gondii* infection in seagull chicks.

In birds, maternal antibodies accumulate in the oocyte during egg yolk formation and receptor-mediated absorption by the chick begins shortly before hatching [29]. However, these antibodies are usually considered to decay within few days or weeks after hatching in classical model species, such as quails and chickens [14]. In another gull species, the black-legged kittiwake (*Rissa trydactila*), the rate of decay is high and levels of antibodies are very low two weeks after hatching [30]. In our study, most yellow legged-gull chicks were sampled when they were older than ten days old. Moreover, if the antibodies of young seropositive chicks were the result of maternal transfer, seropositivity should decrease with age, but we found the opposite trend. Therefore, the pattern of antibodies in those colonies indicates that the main route of transmission of *T*. *gondii* in seagulls is horizontal as the exposure to the parasite occurs through the feeding of chicks. In addition, it has to be taken into account that at least in chickens, infection in hens very rarely results in egg infection, and when egg infection occurs, it generally produces egg mortality and clinical toxoplasmosis in chicks [31], thus preventing vertical infection to be successful.

To our knowledge, *T*. *gondii* has been isolated from black-headed gulls (*L*. *ridibundus*), while antibodies have been reported in ring-billed gulls (*L*. *delawarensis*) and in laughing gulls (*L*. *atricilla*) [1,32]. The overall seroprevalence observed in the present study is in line with that found in black-headed gulls (19.9% of 659) in China using the same diagnosis method but with a cut off titer of ≥ 1:5 [32]. Previous reports showed seroprevalence levels of 15.3% in 13 ring-billed gull and 6% in 33 laughing gulls [1]. Negative results were observed in 127 gulls (*Larus* spp.) from Norway and Sweden [33], and in 27 glaucous gulls (*L*. *hyperboreus*) from Svalbard Island in the high Arctic [11]. However, differences in seroprevalence among studies have to be compared carefully since techniques, species, geographical and/or climatological conditions varied among studies. Our results indicate widespread exposure of *T*. *gondii* in seagulls in the Iberian Peninsula, in agreement with previous studies in many wild bird species in the South of Europe [5,6,7,21,34].

As other intermediate hosts, seagulls can be infected with *T*. *gondii* by ingestion of sporulated oocysts, ingestion of bradyzoites in tissue cysts of other intermediate hosts or vertically. *Toxoplasma gondii* oocysts from cat feces are believed to be washed into sewage and fresh-water runoffs to the marine ecosystems [4], where oocysts remain infective for up to 24 months [35]. In the aquatic ecosystems, oocysts have proven to be accumulated by filter-feeding invertebrates or fishes [36,37], and that has been suggested as the route of transmission by which aquatic ecosystems are linked to warm-blooded animals. [3,38]. Also, high concentrations of oocysts of the parasite in water were also positively correlated with the proximity of human settlements and sewages [4].

In a recent study carried out in a large number of wild bird species with a large number of samples analyzed, the main risk factors associated with *T*. *gondii* seropositivity in wild birds in Spain were age and feeding behavior [5]. The diet reconstruction shows, as expected, a heavy dependence on marine sources for the colonies closer to important fishing grounds (Columbretes, Ons and Ebro Delta). In the particular case of the Ebro Delta, in which gulls have easy access to brackish and freshwater environments, freshwater prey has a relatively high importance as a secondary food source. In Medes Islands fisheries discards are not abundant, but the Empordà wetlands nearby provide a constant and predictable source of freshwater prey, and this is certainly reflected in the diet reconstruction. The opportunistic nature of this species is reflected in the changes in diet in the Dragonera colony after the covering of the local landfill reduced the availability of dumpsite-related food sources, and a rodent extermination intensive program that poisoned a great number of birds and drastically reduced the population [39]. Interestingly, our results detected the highest seroprevalence in colonies that had freshwater as their primary food source. Most of the seagull colonies in our study had access to the shore and coastal aquatic areas close to human settlements, which could increase the risk of exposure to this parasite [1]. The lowest seroprevalence in yellow-legged gulls was observed in the colonies from Ons Island, located in the Atlantic Ocean, where the main source of food was marine followed by refuse, and where freshwater and terrestrial food origin represented only a small percentage [17]. On the contrary, the highest seroprevalence was observed in the Mediterranean island of Medes with a freshwater source of food as the main diet. There was also higher prevalence of antibodies in the chicks that had marine as the secondary source of food. We can assume that the presence of *T*. *gondii* oocysts was high in filter feeding invertebrates or fish provided to the seagull chicks of our study during the first weeks of life. These data would indicate the aquatic environment as one of the main routes of transport and accumulation of *T*. *gondii* oocysts in the Mediterranean basin. This hypothesis is in agreement with previously reported *T*. *gondii* infection in several dolphin species from the Western Mediterranean sea [40,41].

The assessment of *T*. *gondii* infection in seagulls confirms the susceptibility to *T*. *gondii* infection and points these bird species as intermediate hosts. Therefore, as subclinical carriers, there would be no health limitations imposed by *T*. *gondii* infection on the seagull species analyzed. In addition, since Audouin´s gull is a migratory species, our results suggest that gulls can act as carriers of the parasite in their migrations, having the capability to introduce pathogens in remote islands as naïve territories and expose to other susceptible hosts. In this sense, our results identify the yellow-legged and Audouin´s gulls as a good sentinel species to monitor the presence of *T*. *gondii* due to their scavenging feeding behavior and the high population densities in certain geographic locations. However, in order to evaluate the role of these species in the dispersion of different *T*. *gondii* biotypes, information on the distance covered by each species during migration and molecular characterization of the parasite in migratory birds would be useful.

## Supporting Information

S1 Table Data about the chick gulls analyzed. Species; MAT: results (positive/negative) of the analysis for the detection of antibodies against *Toxoplasma gondii*; Titer of the antibodies; year of sampling; age (in days) of the analyzed animal; colony of origin; main food source of the chick analyzed.
(DOCX)
