# Supplementary material for: Toxoplasma gondii Infection in Seagull Chicks Is Related to the Consumption of Freshwater Food Resources
Source: PLoS One. 2016 Mar 14;11(3):e0150249. doi: 10.1371/journal.pone.0150249 (PMC4790883; doi:10.1371/journal.pone.0150249)
Supplement: S1 Table — Species; MAT: results (positive/negative) of the analysis for the detection of antibodies against Toxoplasma gondii; Titer of the antibodies; year of sampling; age (in days) of the analyzed animal; colony of origin; main food source of the chick analyzed. (DOCX) [file pone.0150249.s001.docx]

| ID | Species | MAT | TITER | Year | Age (days) | Colony | Food_source |
| --- | --- | --- | --- | --- | --- | --- | --- |
| 1 | Yellow-legged gull | Negative |  | 2010 | 4 | Dragonera island | Marine |
| 2 | Yellow-legged gull | Positive | 50 | 2010 | 5 | Delta de l'Ebre | Marine |
| 3 | Yellow-legged gull | Negative |  | 2010 | 6 | Dragonera island | Marine |
| 4 | Yellow-legged gull | Negative |  | 2010 | 6 | Dragonera island | Marine |
| 5 | Yellow-legged gull | Negative |  | 2010 | 6 | Dragonera island | Marine |
| 6 | Yellow-legged gull | Negative |  | 2010 | 7 | Dragonera island | Marine |
| 7 | Yellow-legged gull | Positive | 50 | 2010 | 7 | Delta de l'Ebre | Marine |
| 8 | Yellow-legged gull | Negative |  | 2010 | 7 | Dragonera island | Marine |
| 9 | Yellow-legged gull | Negative |  | 2010 | 7 | Dragonera island | Marine |
| 10 | Yellow-legged gull | Negative |  | 2011 | 7 | Ons Island | Marine |
| 11 | Yellow-legged gull | Negative |  | 2010 | 7 | Ons Island | Marine |
| 12 | Yellow-legged gull | Negative |  | 2010 | 7 | Delta de l'Ebre | Marine |
| 13 | Yellow-legged gull | Negative |  | 2010 | 7 | Ons Island | Marine |
| 14 | Yellow-legged gull | Negative |  | 2011 | 7 | Ons Island | Marine |
| 15 | Yellow-legged gull | Negative |  | 2010 | 8 | Ons Island | Marine |
| 16 | Yellow-legged gull | Negative |  | 2010 | 8 | Ons Island | Marine |
| 17 | Yellow-legged gull | Negative |  | 2010 | 8 | Dragonera island | Marine |
| 18 | Yellow-legged gull | Negative |  | 2009 | 8 | Delta de l'Ebre | Marine |
| 19 | Yellow-legged gull | Negative |  | 2011 | 8 | Ons Island | Marine |
| 20 | Yellow-legged gull | Negative |  | 2010 | 8 | Dragonera island | Marine |
| 21 | Yellow-legged gull | Negative |  | 2010 | 8 | Dragonera island | Marine |
| 22 | Yellow-legged gull | Negative |  | 2011 | 8 | Ons Island | Marine |
| 23 | Yellow-legged gull | Negative |  | 2011 | 8 | Ons Island | Marine |
| 24 | Yellow-legged gull | Negative |  | 2011 | 8 | Columbretes Islands | Marine |
| 25 | Yellow-legged gull | Negative |  | 2011 | 8 | Dragonera island | Waterfresh |
| 26 | Yellow-legged gull | Negative |  | 2010 | 8 | Delta de l'Ebre | Marine |
| 27 | Yellow-legged gull | Negative |  | 2010 | 8 | Dragonera island | Marine |
| 28 | Yellow-legged gull | Negative |  | 2010 | 9 | Dragonera island | Marine |
| 29 | Yellow-legged gull | Negative |  | 2011 | 9 | Delta de l'Ebre | Marine |
| 30 | Yellow-legged gull | Negative |  | 2010 | 9 | Dragonera island | Marine |
| 31 | Yellow-legged gull | Positive | 25 | 2010 | 9 | Delta de l'Ebre | Marine |
| 32 | Yellow-legged gull | Negative |  | 2009 | 9 | Columbretes Islands | Marine |
| 33 | Yellow-legged gull | Negative |  | 2010 | 9 | Dragonera island | Marine |
| 34 | Yellow-legged gull | Positive | 100 | 2011 | 9 | Ons Island | Marine |
| 35 | Yellow-legged gull | Negative |  | 2010 | 9 | Dragonera island | Marine |
| 36 | Yellow-legged gull | Positive | 50 | 2010 | 9 | Dragonera island | Marine |
| 37 | Yellow-legged gull | Negative |  | 2010 | 10 | Delta de l'Ebre | Marine |
| 38 | Yellow-legged gull | Negative |  | 2010 | 10 | Ons Island | Marine |
| 39 | Yellow-legged gull | Negative |  | 2011 | 10 | Columbretes Islands | Marine |
| 40 | Yellow-legged gull | Negative |  | 2011 | 10 | Dragonera island | Waterfresh |
| 41 | Yellow-legged gull | Negative |  | 2011 | 10 | Dragonera island | Waterfresh |
| 42 | Yellow-legged gull | Negative |  | 2011 | 10 | Medas Islands | Waterfresh |
| 43 | Yellow-legged gull | Negative |  | 2011 | 10 | Delta de l'Ebre | Marine |
| 44 | Yellow-legged gull | Negative |  | 2011 | 10 | Columbretes Islands | Marine |
| 45 | Yellow-legged gull | Positive |  | 2009 | 10 | Columbretes Islands | Marine |
| 46 | Yellow-legged gull | Negative |  | 2011 | 10 | Delta de l'Ebre | Marine |
| 47 | Yellow-legged gull | Negative |  | 2011 | 10 | Columbretes Islands | Marine |
| 48 | Yellow-legged gull | Negative |  | 2010 | 10 | Ons Island | Marine |
| 49 | Yellow-legged gull | Negative |  | 2011 | 10 | Dragonera island | Waterfresh |
| 50 | Yellow-legged gull | Negative |  | 2010 | 10 | Delta de l'Ebre | Marine |
| 51 | Yellow-legged gull | Negative |  | 2010 | 11 | Delta de l'Ebre | Marine |
| 52 | Yellow-legged gull | Negative |  | 2011 | 11 | Dragonera island | Waterfresh |
| 53 | Yellow-legged gull | Negative |  | 2010 | 11 | Columbretes Islands | Marine |
| 54 | Yellow-legged gull | Negative |  | 2010 | 11 | Ons Island | Marine |
| 55 | Yellow-legged gull | Negative |  | 2010 | 11 | Dragonera island | Marine |
| 56 | Yellow-legged gull | Negative |  | 2011 | 11 | Ons Island | Marine |
| 57 | Yellow-legged gull | Negative |  | 2011 | 11 | Ons Island | Marine |
| 58 | Yellow-legged gull | Negative |  | 2011 | 11 | Medas Islands | Waterfresh |
| 59 | Yellow-legged gull | Negative |  | 2011 | 11 | Columbretes Islands | Marine |
| 60 | Yellow-legged gull | Negative |  | 2010 | 11 | Delta de l'Ebre | Marine |
| 61 | Yellow-legged gull | Negative |  | 2010 | 11 | Delta de l'Ebre | Marine |
| 62 | Yellow-legged gull | Negative |  | 2010 | 11 | Dragonera island | Marine |
| 63 | Yellow-legged gull | Negative |  | 2011 | 11 | Ons Island | Marine |
| 64 | Yellow-legged gull | Negative |  | 2010 | 12 | Ons Island | Marine |
| 65 | Yellow-legged gull | Positive | 25 | 2010 | 12 | Delta de l'Ebre | Marine |
| 66 | Yellow-legged gull | Negative |  | 2011 | 12 | Delta de l'Ebre | Marine |
| 67 | Yellow-legged gull | Negative |  | 2010 | 12 | Ons Island | Marine |
| 68 | Yellow-legged gull | Negative |  | 2010 | 12 | Dragonera island | Marine |
| 69 | Yellow-legged gull | Negative |  | 2011 | 12 | Delta de l'Ebre | Marine |
| 70 | Yellow-legged gull | Negative |  | 2011 | 12 | Delta de l'Ebre | Marine |
| 71 | Yellow-legged gull | Positive |  | 2009 | 12 | Columbretes Islands | Marine |
| 72 | Yellow-legged gull | Negative |  | 2010 | 12 | Delta de l'Ebre | Marine |
| 73 | Yellow-legged gull | Negative |  | 2011 | 12 | Dragonera island | Waterfresh |
| 74 | Yellow-legged gull | Negative |  | 2011 | 12 | Ons Island | Marine |
| 75 | Yellow-legged gull | Negative |  | 2011 | 12 | Delta de l'Ebre | Marine |
| 76 | Yellow-legged gull | Negative |  | 2010 | 12 | Dragonera island | Marine |
| 77 | Yellow-legged gull | Negative |  | 2011 | 12 | Ons Island | Marine |
| 78 | Yellow-legged gull | Negative |  | 2011 | 12 | Columbretes Islands | Marine |
| 79 | Yellow-legged gull | Negative |  | 2010 | 12 | Dragonera island | Marine |
| 80 | Yellow-legged gull | Negative |  | 2010 | 12 | Delta de l'Ebre | Marine |
| 81 | Yellow-legged gull | Negative |  | 2010 | 12 | Dragonera island | Marine |
| 82 | Yellow-legged gull | Negative |  | 2010 | 12 | Ons Island | Marine |
| 83 | Yellow-legged gull | Negative |  | 2011 | 12 | Ons Island | Marine |
| 84 | Yellow-legged gull | Negative |  | 2011 | 12 | Delta de l'Ebre | Marine |
| 85 | Yellow-legged gull | Negative |  | 2010 | 12 | Ons Island | Marine |
| 86 | Yellow-legged gull | Positive | 25 | 2010 | 12 | Ons Island | Marine |
| 87 | Yellow-legged gull | Negative |  | 2011 | 12 | Dragonera island | Waterfresh |
| 88 | Yellow-legged gull | Negative |  | 2011 | 12 | Ons Island | Marine |
| 89 | Yellow-legged gull | Negative |  | 2009 | 13 | Columbretes Islands | Marine |
| 90 | Yellow-legged gull | Negative |  | 2010 | 13 | Delta de l'Ebre | Marine |
| 91 | Yellow-legged gull | Negative |  | 2010 | 13 | Ons Island | Marine |
| 92 | Yellow-legged gull | Negative |  | 2011 | 13 | Ons Island | Marine |
| 93 | Yellow-legged gull | Negative |  | 2011 | 13 | Columbretes Islands | Marine |
| 94 | Yellow-legged gull | Negative |  | 2010 | 13 | Dragonera island | Marine |
| 95 | Yellow-legged gull | Negative |  | 2010 | 13 | Ons Island | Marine |
| 96 | Yellow-legged gull | Negative |  | 2010 | 13 | Ons Island | Marine |
| 97 | Yellow-legged gull | Negative |  | 2011 | 13 | Medas Islands | Waterfresh |
| 98 | Yellow-legged gull | Negative |  | 2009 | 13 | Columbretes Islands | Marine |
| 99 | Yellow-legged gull | Negative |  | 2010 | 13 | Delta de l'Ebre | Marine |
| 100 | Yellow-legged gull | Positive | 50 | 2011 | 13 | Medas Islands | Waterfresh |
| 101 | Yellow-legged gull | Negative |  | 2010 | 13 | Delta de l'Ebre | Marine |
| 102 | Yellow-legged gull | Negative |  | 2011 | 13 | Delta de l'Ebre | Marine |
| 103 | Yellow-legged gull | Negative |  | 2011 | 13 | Delta de l'Ebre | Marine |
| 104 | Yellow-legged gull | Positive | 50 | 2011 | 13 | Medas Islands | Waterfresh |
| 105 | Yellow-legged gull | Positive | 25 | 2011 | 13 | Medas Islands | Waterfresh |
| 106 | Yellow-legged gull | Negative |  | 2011 | 13 | Delta de l'Ebre | Marine |
| 107 | Yellow-legged gull | Negative |  | 2011 | 13 | Ons Island | Marine |
| 108 | Yellow-legged gull | Negative |  | 2011 | 13 | Medas Islands | Waterfresh |
| 109 | Yellow-legged gull | Negative |  | 2011 | 13 | Dragonera island | Waterfresh |
| 110 | Yellow-legged gull | Negative |  | 2011 | 13 | Delta de l'Ebre | Marine |
| 111 | Yellow-legged gull | Negative |  | 2011 | 13 | Ons Island | Marine |
| 112 | Yellow-legged gull | Negative |  | 2010 | 13 | Ons Island | Marine |
| 113 | Yellow-legged gull | Negative |  | 2011 | 13 | Dragonera island | Waterfresh |
| 114 | Yellow-legged gull | Negative |  | 2011 | 13 | Delta de l'Ebre | Marine |
| 115 | Yellow-legged gull | Negative |  | 2011 | 13 | Ons Island | Marine |
| 116 | Yellow-legged gull | Negative |  | 2010 | 13 | Dragonera island | Marine |
| 117 | Yellow-legged gull | Negative |  | 2011 | 13 | Ons Island | Marine |
| 118 | Yellow-legged gull | Negative |  | 2009 | 14 | Columbretes Islands | Marine |
| 119 | Yellow-legged gull | Negative |  | 2011 | 14 | Ons Island | Marine |
| 120 | Yellow-legged gull | Negative |  | 2011 | 14 | Dragonera island | Waterfresh |
| 121 | Yellow-legged gull | Negative |  | 2011 | 14 | Medas Islands | Waterfresh |
| 122 | Yellow-legged gull | Negative |  | 2011 | 14 | Ons Island | Marine |
| 123 | Yellow-legged gull | Negative |  | 2010 | 14 | Ons Island | Marine |
| 124 | Yellow-legged gull | Negative |  | 2011 | 14 | Ons Island | Marine |
| 125 | Yellow-legged gull | Positive |  | 2009 | 14 | Columbretes Islands | Marine |
| 126 | Yellow-legged gull | Negative |  | 2011 | 14 | Ons Island | Marine |
| 127 | Yellow-legged gull | Positive | 50 | 2011 | 14 | Dragonera island | Waterfresh |
| 128 | Yellow-legged gull | Negative |  | 2011 | 14 | Ons Island | Marine |
| 129 | Yellow-legged gull | Negative |  | 2011 | 14 | Ons Island | Marine |
| 130 | Yellow-legged gull | Negative |  | 2011 | 14 | Dragonera island | Waterfresh |
| 131 | Yellow-legged gull | Negative |  | 2011 | 14 | Dragonera island | Waterfresh |
| 132 | Yellow-legged gull | Positive |  | 2009 | 14 | Columbretes Islands | Marine |
| 133 | Yellow-legged gull | Negative |  | 2010 | 14 | Delta de l'Ebre | Marine |
| 134 | Yellow-legged gull | Negative |  | 2011 | 14 | Ons Island | Marine |
| 135 | Yellow-legged gull | Negative |  | 2009 | 14 | Columbretes Islands | Marine |
| 136 | Yellow-legged gull | Negative |  | 2011 | 14 | Delta de l'Ebre | Marine |
| 137 | Yellow-legged gull | Negative |  | 2011 | 14 | Medas Islands | Waterfresh |
| 138 | Yellow-legged gull | Negative |  | 2010 | 14 | Ons Island | Marine |
| 139 | Yellow-legged gull | Negative |  | 2011 | 14 | Delta de l'Ebre | Marine |
| 140 | Yellow-legged gull | Negative |  | 2010 | 14 | Dragonera island | Marine |
| 141 | Yellow-legged gull | Positive | 100 | 2011 | 14 | Dragonera island | Waterfresh |
| 142 | Yellow-legged gull | Negative |  | 2010 | 14 | Ons Island | Marine |
| 143 | Yellow-legged gull | Negative |  | 2011 | 14 | Dragonera island | Waterfresh |
| 144 | Yellow-legged gull | Negative |  | 2009 | 14 | Columbretes Islands | Marine |
| 145 | Yellow-legged gull | Negative |  | 2011 | 15 | Ons Island | Marine |
| 146 | Yellow-legged gull | Negative |  | 2010 | 15 | Columbretes Islands | Marine |
| 147 | Yellow-legged gull | Positive | 25 | 2011 | 15 | Medas Islands | Waterfresh |
| 148 | Yellow-legged gull | Positive | 25 | 2011 | 15 | Medas Islands | Waterfresh |
| 149 | Yellow-legged gull | Negative |  | 2011 | 15 | Dragonera island | Waterfresh |
| 150 | Yellow-legged gull | Positive | 50 | 2011 | 15 | Dragonera island | Waterfresh |
| 151 | Yellow-legged gull | Negative |  | 2010 | 15 | Columbretes Islands | Marine |
| 152 | Yellow-legged gull | Negative |  | 2011 | 15 | Delta de l'Ebre | Marine |
| 153 | Yellow-legged gull | Negative |  | 2011 | 15 | Dragonera island | Waterfresh |
| 154 | Yellow-legged gull | Negative |  | 2011 | 15 | Delta de l'Ebre | Marine |
| 155 | Yellow-legged gull | Negative |  | 2011 | 15 | Ons Island | Marine |
| 156 | Yellow-legged gull | Negative |  | 2011 | 15 | Columbretes Islands | Marine |
| 157 | Yellow-legged gull | Negative |  | 2011 | 15 | Ons Island | Marine |
| 158 | Yellow-legged gull | Negative |  | 2011 | 15 | Dragonera island | Waterfresh |
| 159 | Yellow-legged gull | Negative |  | 2011 | 15 | Delta de l'Ebre | Marine |
| 160 | Yellow-legged gull | Negative |  | 2009 | 15 | Columbretes Islands | Marine |
| 161 | Yellow-legged gull | Negative |  | 2009 | 15 | Delta de l'Ebre | Marine |
| 162 | Yellow-legged gull | Negative |  | 2010 | 15 | Delta de l'Ebre | Marine |
| 163 | Yellow-legged gull | Negative |  | 2011 | 15 | Medas Islands | Waterfresh |
| 164 | Yellow-legged gull | Negative |  | 2010 | 15 | Columbretes Islands | Marine |
| 165 | Yellow-legged gull | Negative |  | 2011 | 15 | Medas Islands | Waterfresh |
| 166 | Yellow-legged gull | Negative |  | 2010 | 15 | Ons Island | Marine |
| 167 | Yellow-legged gull | Negative |  | 2011 | 15 | Ons Island | Marine |
| 168 | Yellow-legged gull | Negative |  | 2010 | 15 | Delta de l'Ebre | Marine |
| 169 | Yellow-legged gull | Negative |  | 2011 | 15 | Delta de l'Ebre | Marine |
| 170 | Yellow-legged gull | Positive |  | 2009 | 16 | Columbretes Islands | Marine |
| 171 | Yellow-legged gull | Negative |  | 2011 | 16 | Medas Islands | Waterfresh |
| 172 | Yellow-legged gull | Negative |  | 2010 | 16 | Dragonera island | Marine |
| 173 | Yellow-legged gull | Negative |  | 2011 | 16 | Delta de l'Ebre | Marine |
| 174 | Yellow-legged gull | Negative |  | 2011 | 16 | Ons Island | Marine |
| 175 | Yellow-legged gull | Negative |  | 2011 | 16 | Dragonera island | Waterfresh |
| 176 | Yellow-legged gull | Negative |  | 2011 | 16 | Delta de l'Ebre | Marine |
| 177 | Yellow-legged gull | Negative |  | 2011 | 16 | Columbretes Islands | Marine |
| 178 | Yellow-legged gull | Negative |  | 2009 | 16 | Medas Islands | Waterfresh |
| 179 | Yellow-legged gull | Positive | 25 | 2009 | 16 | Medas Islands | Waterfresh |
| 180 | Yellow-legged gull | Negative |  | 2010 | 16 | Dragonera island | Marine |
| 181 | Yellow-legged gull | Negative |  | 2011 | 16 | Delta de l'Ebre | Marine |
| 182 | Yellow-legged gull | Negative |  | 2011 | 16 | Delta de l'Ebre | Marine |
| 183 | Yellow-legged gull | Negative |  | 2009 | 16 | Columbretes Islands | Marine |
| 184 | Yellow-legged gull | Negative |  | 2010 | 16 | Columbretes Islands | Marine |
| 185 | Yellow-legged gull | Negative |  | 2011 | 16 | Dragonera island | Waterfresh |
| 186 | Yellow-legged gull | Negative |  | 2011 | 16 | Delta de l'Ebre | Marine |
| 187 | Yellow-legged gull | Negative |  | 2011 | 16 | Medas Islands | Waterfresh |
| 188 | Yellow-legged gull | Negative |  | 2010 | 16 | Delta de l'Ebre | Marine |
| 189 | Yellow-legged gull | Negative |  | 2011 | 16 | Delta de l'Ebre | Marine |
| 190 | Yellow-legged gull | Negative |  | 2011 | 16 | Dragonera island | Waterfresh |
| 191 | Yellow-legged gull | Negative |  | 2010 | 16 | Delta de l'Ebre | Marine |
| 192 | Yellow-legged gull | Negative |  | 2010 | 16 | Ons Island | Marine |
| 193 | Yellow-legged gull | Negative |  | 2011 | 16 | Delta de l'Ebre | Marine |
| 194 | Yellow-legged gull | Positive |  | 2009 | 16 | Columbretes Islands | Marine |
| 195 | Yellow-legged gull | Negative |  | 2011 | 16 | Dragonera island | Waterfresh |
| 196 | Yellow-legged gull | Negative |  | 2010 | 16 | Delta de l'Ebre | Marine |
| 197 | Yellow-legged gull | Negative |  | 2011 | 16 | Dragonera island | Waterfresh |
| 198 | Yellow-legged gull | Negative |  | 2009 | 16 | Columbretes Islands | Marine |
| 199 | Yellow-legged gull | Negative |  | 2010 | 16 | Dragonera island | Marine |
| 200 | Yellow-legged gull | Negative |  | 2010 | 16 | Ons Island | Marine |
| 201 | Yellow-legged gull | Negative |  | 2011 | 16 | Columbretes Islands | Marine |
| 202 | Yellow-legged gull | Negative |  | 2010 | 16 | Delta de l'Ebre | Marine |
| 203 | Yellow-legged gull | Positive | 25 | 2010 | 16 | Delta de l'Ebre | Marine |
| 204 | Yellow-legged gull | Positive | 25 | 2011 | 16 | Medas Islands | Waterfresh |
| 205 | Yellow-legged gull | Negative |  | 2011 | 16 | Columbretes Islands | Marine |
| 206 | Yellow-legged gull | Negative |  | 2010 | 16 | Delta de l'Ebre | Marine |
| 207 | Yellow-legged gull | Negative |  | 2010 | 16 | Medas Islands | Waterfresh |
| 208 | Yellow-legged gull | Positive | 25 | 2009 | 16 | Medas Islands | Waterfresh |
| 209 | Yellow-legged gull | Negative |  | 2010 | 16 | Ons Island | Marine |
| 210 | Yellow-legged gull | Negative |  | 2011 | 16 | Dragonera island | Waterfresh |
| 211 | Yellow-legged gull | Negative |  | 2010 | 16 | Columbretes Islands | Marine |
| 212 | Yellow-legged gull | Negative |  | 2010 | 17 | Medas Islands | Waterfresh |
| 213 | Yellow-legged gull | Negative |  | 2011 | 17 | Delta de l'Ebre | Marine |
| 214 | Yellow-legged gull | Positive | 25 | 2010 | 17 | Delta de l'Ebre | Marine |
| 215 | Yellow-legged gull | Negative |  | 2011 | 17 | Medas Islands | Waterfresh |
| 216 | Yellow-legged gull | Negative |  | 2011 | 17 | Medas Islands | Waterfresh |
| 217 | Yellow-legged gull | Negative |  | 2010 | 17 | Ons Island | Marine |
| 218 | Yellow-legged gull | Negative |  | 2011 | 17 | Delta de l'Ebre | Marine |
| 219 | Yellow-legged gull | Positive | 100 | 2010 | 17 | Medas Islands | Waterfresh |
| 220 | Yellow-legged gull | Negative |  | 2011 | 17 | Delta de l'Ebre | Marine |
| 221 | Yellow-legged gull | Negative |  | 2011 | 17 | Medas Islands | Waterfresh |
| 222 | Yellow-legged gull | Positive |  | 2009 | 17 | Columbretes Islands | Marine |
| 223 | Yellow-legged gull | Negative |  | 2011 | 17 | Delta de l'Ebre | Marine |
| 224 | Yellow-legged gull | Positive | 50 | 2010 | 17 | Medas Islands | Waterfresh |
| 225 | Yellow-legged gull | Negative |  | 2011 | 17 | Dragonera island | Waterfresh |
| 226 | Yellow-legged gull | Negative |  | 2011 | 17 | Dragonera island | Waterfresh |
| 227 | Yellow-legged gull | Negative |  | 2010 | 17 | Delta de l'Ebre | Marine |
| 228 | Yellow-legged gull | Negative |  | 2010 | 17 | Dragonera island | Marine |
| 229 | Yellow-legged gull | Negative |  | 2011 | 17 | Medas Islands | Waterfresh |
| 230 | Yellow-legged gull | Negative |  | 2010 | 17 | Ons Island | Marine |
| 231 | Yellow-legged gull | Negative |  | 2011 | 17 | Dragonera island | Waterfresh |
| 232 | Yellow-legged gull | Negative |  | 2011 | 17 | Medas Islands | Waterfresh |
| 233 | Yellow-legged gull | Negative |  | 2009 | 17 | Columbretes Islands | Marine |
| 234 | Yellow-legged gull | Negative |  | 2011 | 17 | Columbretes Islands | Marine |
| 235 | Yellow-legged gull | Negative |  | 2009 | 17 | Columbretes Islands | Marine |
| 236 | Yellow-legged gull | Negative |  | 2009 | 17 | Delta de l'Ebre | Marine |
| 237 | Yellow-legged gull | Positive | 25 | 2009 | 17 | Medas Islands | Waterfresh |
| 238 | Yellow-legged gull | Negative |  | 2011 | 17 | Medas Islands | Waterfresh |
| 239 | Yellow-legged gull | Negative |  | 2010 | 17 | Columbretes Islands | Marine |
| 240 | Yellow-legged gull | Negative |  | 2010 | 17 | Medas Islands | Waterfresh |
| 241 | Yellow-legged gull | Negative |  | 2010 | 17 | Dragonera island | Marine |
| 242 | Yellow-legged gull | Negative |  | 2011 | 18 | Dragonera island | Waterfresh |
| 243 | Yellow-legged gull | Positive | 50 | 2011 | 18 | Medas Islands | Waterfresh |
| 244 | Yellow-legged gull | Negative |  | 2009 | 18 | Medas Islands | Waterfresh |
| 245 | Yellow-legged gull | Negative |  | 2011 | 18 | Delta de l'Ebre | Marine |
| 246 | Yellow-legged gull | Negative |  | 2009 | 18 | Delta de l'Ebre | Marine |
| 247 | Yellow-legged gull | Negative |  | 2010 | 18 | Columbretes Islands | Marine |
| 248 | Yellow-legged gull | Positive |  | 2009 | 18 | Columbretes Islands | Marine |
| 249 | Yellow-legged gull | Negative |  | 2011 | 18 | Delta de l'Ebre | Marine |
| 250 | Yellow-legged gull | Negative |  | 2009 | 18 | Delta de l'Ebre | Marine |
| 251 | Yellow-legged gull | Negative |  | 2010 | 18 | Medas Islands | Waterfresh |
| 252 | Yellow-legged gull | Positive | 50 | 2011 | 18 | Columbretes Islands | Marine |
| 253 | Yellow-legged gull | Negative |  | 2011 | 18 | Columbretes Islands | Marine |
| 254 | Yellow-legged gull | Negative |  | 2009 | 18 | Columbretes Islands | Marine |
| 255 | Yellow-legged gull | Negative |  | 2009 | 18 | Columbretes Islands | Marine |
| 256 | Yellow-legged gull | Negative |  | 2010 | 18 | Medas Islands | Waterfresh |
| 257 | Yellow-legged gull | Positive | 50 | 2011 | 18 | Medas Islands | Waterfresh |
| 258 | Yellow-legged gull | Negative |  | 2011 | 18 | Ons Island | Marine |
| 259 | Yellow-legged gull | Positive | 100 | 2011 | 18 | Ons Island | Marine |
| 260 | Yellow-legged gull | Negative |  | 2011 | 18 | Columbretes Islands | Marine |
| 261 | Yellow-legged gull | Negative |  | 2010 | 18 | Delta de l'Ebre | Marine |
| 262 | Yellow-legged gull | Negative |  | 2009 | 18 | Columbretes Islands | Marine |
| 263 | Yellow-legged gull | Positive | 100 | 2010 | 18 | Delta de l'Ebre | Marine |
| 264 | Yellow-legged gull | Negative |  | 2011 | 18 | Delta de l'Ebre | Marine |
| 265 | Yellow-legged gull | Negative |  | 2011 | 18 | Ons Island | Marine |
| 266 | Yellow-legged gull | Negative |  | 2010 | 18 | Medas Islands | Waterfresh |
| 267 | Yellow-legged gull | Negative |  | 2010 | 18 | Columbretes Islands | Marine |
| 268 | Yellow-legged gull | Positive | 50 | 2010 | 18 | Medas Islands | Waterfresh |
| 269 | Yellow-legged gull | Negative |  | 2011 | 18 | Dragonera island | Waterfresh |
| 270 | Yellow-legged gull | Negative |  | 2009 | 18 | Delta de l'Ebre | Marine |
| 271 | Yellow-legged gull | Negative |  | 2010 | 19 | Medas Islands | Waterfresh |
| 272 | Yellow-legged gull | Positive |  | 2009 | 19 | Columbretes Islands | Marine |
| 273 | Yellow-legged gull | Positive |  | 2009 | 19 | Columbretes Islands | Marine |
| 274 | Yellow-legged gull | Negative |  | 2010 | 19 | Dragonera island | Marine |
| 275 | Yellow-legged gull | Negative |  | 2011 | 19 | Dragonera island | Waterfresh |
| 276 | Yellow-legged gull | Negative |  | 2009 | 19 | Medas Islands | Waterfresh |
| 277 | Yellow-legged gull | Positive | 25 | 2009 | 19 | Medas Islands | Waterfresh |
| 278 | Yellow-legged gull | Positive | 25 | 2009 | 19 | Medas Islands | Waterfresh |
| 279 | Yellow-legged gull | Negative |  | 2010 | 19 | Medas Islands | Waterfresh |
| 280 | Yellow-legged gull | Negative |  | 2010 | 19 | Columbretes Islands | Marine |
| 281 | Yellow-legged gull | Negative |  | 2011 | 19 | Ons Island | Marine |
| 282 | Yellow-legged gull | Positive | 25 | 2011 | 19 | Medas Islands | Waterfresh |
| 283 | Yellow-legged gull | Negative |  | 2009 | 19 | Columbretes Islands | Marine |
| 284 | Yellow-legged gull | Positive |  | 2009 | 19 | Columbretes Islands | Marine |
| 285 | Yellow-legged gull | Negative |  | 2010 | 19 | Ons Island | Marine |
| 286 | Yellow-legged gull | Negative |  | 2010 | 19 | Ons Island | Marine |
| 287 | Yellow-legged gull | Negative |  | 2009 | 19 | Columbretes Islands | Marine |
| 288 | Yellow-legged gull | Positive |  | 2009 | 19 | Columbretes Islands | Marine |
| 289 | Yellow-legged gull | Negative |  | 2009 | 19 | Delta de l'Ebre | Marine |
| 290 | Yellow-legged gull | Negative |  | 2010 | 19 | Columbretes Islands | Marine |
| 291 | Yellow-legged gull | Negative |  | 2009 | 19 | Delta de l'Ebre | Marine |
| 292 | Yellow-legged gull | Negative |  | 2011 | 19 | Dragonera island | Waterfresh |
| 293 | Yellow-legged gull | Negative |  | 2010 | 19 | Ons Island | Marine |
| 294 | Yellow-legged gull | Positive | 50 | 2011 | 19 | Columbretes Islands | Marine |
| 295 | Yellow-legged gull | Negative |  | 2010 | 19 | Medas Islands | Waterfresh |
| 296 | Yellow-legged gull | Negative |  | 2010 | 19 | Medas Islands | Waterfresh |
| 297 | Yellow-legged gull | Positive | 25 | 2011 | 19 | Columbretes Islands | Marine |
| 298 | Yellow-legged gull | Negative |  | 2011 | 19 | Delta de l'Ebre | Marine |
| 299 | Yellow-legged gull | Negative |  | 2009 | 19 | Columbretes Islands | Marine |
| 300 | Yellow-legged gull | Positive |  | 2009 | 19 | Columbretes Islands | Marine |
| 301 | Yellow-legged gull | Negative |  | 2010 | 19 | Columbretes Islands | Marine |
| 302 | Yellow-legged gull | Negative |  | 2011 | 19 | Delta de l'Ebre | Marine |
| 303 | Yellow-legged gull | Negative |  | 2011 | 19 | Columbretes Islands | Marine |
| 304 | Yellow-legged gull | Negative |  | 2009 | 19 | Delta de l'Ebre | Marine |
| 305 | Yellow-legged gull | Negative |  | 2011 | 19 | Medas Islands | Waterfresh |
| 306 | Yellow-legged gull | Negative |  | 2011 | 19 | Columbretes Islands | Marine |
| 307 | Yellow-legged gull | Positive | 500 | 2010 | 19 | Columbretes Islands | Marine |
| 308 | Yellow-legged gull | Negative |  | 2011 | 19 | Dragonera island | Waterfresh |
| 309 | Yellow-legged gull | Positive | 50 | 2011 | 19 | Dragonera island | Waterfresh |
| 310 | Yellow-legged gull | Negative |  | 2011 | 19 | Columbretes Islands | Marine |
| 311 | Yellow-legged gull | Negative |  | 2010 | 19 | Columbretes Islands | Marine |
| 312 | Yellow-legged gull | Negative |  | 2009 | 19 | Delta de l'Ebre | Marine |
| 313 | Yellow-legged gull | Positive |  | 2009 | 20 | Columbretes Islands | Marine |
| 314 | Yellow-legged gull | Positive | 25 | 2011 | 20 | Medas Islands | Waterfresh |
| 315 | Yellow-legged gull | Positive | 100 | 2011 | 20 | Ons Island | Marine |
| 316 | Yellow-legged gull | Positive |  | 2009 | 20 | Columbretes Islands | Marine |
| 317 | Yellow-legged gull | Negative |  | 2010 | 20 | Columbretes Islands | Marine |
| 318 | Yellow-legged gull | Negative |  | 2010 | 20 | Medas Islands | Waterfresh |
| 319 | Yellow-legged gull | Positive | 25 | 2011 | 20 | Medas Islands | Waterfresh |
| 320 | Yellow-legged gull | Negative |  | 2009 | 20 | Columbretes Islands | Marine |
| 321 | Yellow-legged gull | Positive |  | 2009 | 20 | Columbretes Islands | Marine |
| 322 | Yellow-legged gull | Negative |  | 2010 | 20 | Medas Islands | Waterfresh |
| 323 | Yellow-legged gull | Negative |  | 2011 | 20 | Columbretes Islands | Marine |
| 324 | Yellow-legged gull | Negative |  | 2009 | 20 | Columbretes Islands | Marine |
| 325 | Yellow-legged gull | Negative |  | 2011 | 20 | Columbretes Islands | Marine |
| 326 | Yellow-legged gull | Negative |  | 2010 | 20 | Columbretes Islands | Marine |
| 327 | Yellow-legged gull | Negative |  | 2009 | 20 | Delta de l'Ebre | Marine |
| 328 | Yellow-legged gull | Positive | 25 | 2010 | 20 | Delta de l'Ebre | Marine |
| 329 | Yellow-legged gull | Negative |  | 2009 | 20 | Medas Islands | Waterfresh |
| 330 | Yellow-legged gull | Negative |  | 2011 | 20 | Medas Islands | Waterfresh |
| 331 | Yellow-legged gull | Positive | 50 | 2010 | 20 | Delta de l'Ebre | Marine |
| 332 | Yellow-legged gull | Negative |  | 2010 | 20 | Medas Islands | Waterfresh |
| 333 | Yellow-legged gull | Negative |  | 2011 | 20 | Ons Island | Marine |
| 334 | Yellow-legged gull | Negative |  | 2010 | 20 | Columbretes Islands | Marine |
| 335 | Yellow-legged gull | Positive | 50 | 2009 | 20 | Medas Islands | Waterfresh |
| 336 | Yellow-legged gull | Negative |  | 2010 | 20 | Ons Island | Marine |
| 337 | Yellow-legged gull | Negative |  | 2009 | 20 | Delta de l'Ebre | Marine |
| 338 | Yellow-legged gull | Negative |  | 2011 | 20 | Delta de l'Ebre | Marine |
| 339 | Yellow-legged gull | Negative |  | 2010 | 20 | Ons Island | Marine |
| 340 | Yellow-legged gull | Negative |  | 2009 | 20 | Columbretes Islands | Marine |
| 341 | Yellow-legged gull | Negative |  | 2009 | 20 | Columbretes Islands | Marine |
| 342 | Yellow-legged gull | Negative |  | 2010 | 20 | Columbretes Islands | Marine |
| 343 | Yellow-legged gull | Positive | 100 | 2010 | 20 | Columbretes Islands | Marine |
| 344 | Yellow-legged gull | Negative |  | 2011 | 20 | Columbretes Islands | Marine |
| 345 | Yellow-legged gull | Negative |  | 2010 | 20 | Medas Islands | Waterfresh |
| 346 | Yellow-legged gull | Negative |  | 2010 | 20 | Columbretes Islands | Marine |
| 347 | Yellow-legged gull | Positive | 100 | 2009 | 20 | Delta de l'Ebre | Marine |
| 348 | Yellow-legged gull | Negative |  | 2011 | 21 | Ons Island | Marine |
| 349 | Yellow-legged gull | Negative |  | 2009 | 21 | Medas Islands | Waterfresh |
| 350 | Yellow-legged gull | Negative |  | 2011 | 21 | Columbretes Islands | Marine |
| 351 | Yellow-legged gull | Negative |  | 2011 | 21 | Columbretes Islands | Marine |
| 352 | Yellow-legged gull | Negative |  | 2009 | 21 | Delta de l'Ebre | Marine |
| 353 | Yellow-legged gull | Negative |  | 2009 | 21 | Medas Islands | Waterfresh |
| 354 | Yellow-legged gull | Negative |  | 2009 | 21 | Columbretes Islands | Marine |
| 355 | Yellow-legged gull | Negative |  | 2009 | 21 | Columbretes Islands | Marine |
| 356 | Yellow-legged gull | Positive |  | 2009 | 21 | Columbretes Islands | Marine |
| 357 | Yellow-legged gull | Positive |  | 2009 | 21 | Columbretes Islands | Marine |
| 358 | Yellow-legged gull | Negative |  | 2010 | 21 | Columbretes Islands | Marine |
| 359 | Yellow-legged gull | Negative |  | 2010 | 21 | Ons Island | Marine |
| 360 | Yellow-legged gull | Positive |  | 2009 | 21 | Columbretes Islands | Marine |
| 361 | Yellow-legged gull | Negative |  | 2009 | 21 | Delta de l'Ebre | Marine |
| 362 | Yellow-legged gull | Positive | 50 | 2009 | 21 | Delta de l'Ebre | Marine |
| 363 | Yellow-legged gull | Negative |  | 2009 | 21 | Medas Islands | Waterfresh |
| 364 | Yellow-legged gull | Negative |  | 2009 | 21 | Medas Islands | Waterfresh |
| 365 | Yellow-legged gull | Negative |  | 2011 | 21 | Ons Island | Marine |
| 366 | Yellow-legged gull | Negative |  | 2010 | 21 | Medas Islands | Waterfresh |
| 367 | Yellow-legged gull | Negative |  | 2010 | 21 | Medas Islands | Waterfresh |
| 368 | Yellow-legged gull | Positive |  | 2009 | 21 | Columbretes Islands | Marine |
| 369 | Yellow-legged gull | Negative |  | 2009 | 21 | Medas Islands | Waterfresh |
| 370 | Yellow-legged gull | Negative |  | 2009 | 21 | Medas Islands | Waterfresh |
| 371 | Yellow-legged gull | Negative |  | 2010 | 21 | Medas Islands | Waterfresh |
| 372 | Yellow-legged gull | Negative |  | 2009 | 21 | Columbretes Islands | Marine |
| 373 | Yellow-legged gull | Negative |  | 2009 | 21 | Delta de l'Ebre | Marine |
| 374 | Yellow-legged gull | Positive |  | 2009 | 21 | Columbretes Islands | Marine |
| 375 | Yellow-legged gull | Negative |  | 2010 | 21 | Medas Islands | Waterfresh |
| 376 | Yellow-legged gull | Negative |  | 2010 | 21 | Columbretes Islands | Marine |
| 377 | Yellow-legged gull | Negative |  | 2009 | 22 | Columbretes Islands | Marine |
| 378 | Yellow-legged gull | Positive | 100 | 2009 | 22 | Delta de l'Ebre | Marine |
| 379 | Yellow-legged gull | Positive | 50 | 2009 | 22 | Medas Islands | Waterfresh |
| 380 | Yellow-legged gull | Negative |  | 2011 | 22 | Delta de l'Ebre | Marine |
| 381 | Yellow-legged gull | Negative |  | 2010 | 22 | Columbretes Islands | Marine |
| 382 | Yellow-legged gull | Negative |  | 2009 | 22 | Columbretes Islands | Marine |
| 383 | Yellow-legged gull | Positive |  | 2009 | 22 | Columbretes Islands | Marine |
| 384 | Yellow-legged gull | Positive | 100 | 2009 | 22 | Delta de l'Ebre | Marine |
| 385 | Yellow-legged gull | Negative |  | 2009 | 22 | Medas Islands | Waterfresh |
| 386 | Yellow-legged gull | Positive | 25 | 2011 | 22 | Medas Islands | Waterfresh |
| 387 | Yellow-legged gull | Positive |  | 2009 | 22 | Columbretes Islands | Marine |
| 388 | Yellow-legged gull | Negative |  | 2009 | 22 | Delta de l'Ebre | Marine |
| 389 | Yellow-legged gull | Negative |  | 2011 | 22 | Dragonera island | Waterfresh |
| 390 | Yellow-legged gull | Negative |  | 2009 | 22 | Columbretes Islands | Marine |
| 391 | Yellow-legged gull | Positive | 25 | 2011 | 22 | Medas Islands | Waterfresh |
| 392 | Yellow-legged gull | Negative |  | 2011 | 22 | Dragonera island | Waterfresh |
| 393 | Yellow-legged gull | Negative |  | 2009 | 22 | Delta de l'Ebre | Marine |
| 394 | Yellow-legged gull | Negative |  | 2009 | 22 | Columbretes Islands | Marine |
| 395 | Yellow-legged gull | Negative |  | 2009 | 22 | Columbretes Islands | Marine |
| 396 | Yellow-legged gull | Positive | 50 | 2009 | 22 | Medas Islands | Waterfresh |
| 397 | Yellow-legged gull | Positive |  | 2009 | 22 | Columbretes Islands | Marine |
| 398 | Yellow-legged gull | Negative |  | 2009 | 22 | Columbretes Islands | Marine |
| 399 | Yellow-legged gull | Negative |  | 2009 | 22 | Delta de l'Ebre | Marine |
| 400 | Yellow-legged gull | Negative |  | 2009 | 22 | Medas Islands | Waterfresh |
| 401 | Yellow-legged gull | Positive | 25 | 2010 | 23 | Medas Islands | Waterfresh |
| 402 | Yellow-legged gull | Negative |  | 2009 | 23 | Columbretes Islands | Marine |
| 403 | Yellow-legged gull | Positive |  | 2009 | 23 | Columbretes Islands | Marine |
| 404 | Yellow-legged gull | Positive | 100 | 2010 | 23 | Columbretes Islands | Marine |
| 405 | Yellow-legged gull | Positive | 50 | 2009 | 23 | Delta de l'Ebre | Marine |
| 406 | Yellow-legged gull | Negative |  | 2010 | 23 | Dragonera island | Marine |
| 407 | Yellow-legged gull | Positive | 50 | 2011 | 23 | Medas Islands | Waterfresh |
| 408 | Yellow-legged gull | Negative |  | 2009 | 23 | Columbretes Islands | Marine |
| 409 | Yellow-legged gull | Positive |  | 2009 | 23 | Columbretes Islands | Marine |
| 410 | Yellow-legged gull | Positive | 50 | 2009 | 23 | Medas Islands | Waterfresh |
| 411 | Yellow-legged gull | Negative |  | 2010 | 23 | Medas Islands | Waterfresh |
| 412 | Yellow-legged gull | Negative |  | 2009 | 23 | Delta de l'Ebre | Marine |
| 413 | Yellow-legged gull | Positive | 25 | 2009 | 23 | Delta de l'Ebre | Marine |
| 414 | Yellow-legged gull | Positive | 25 | 2009 | 23 | Medas Islands | Waterfresh |
| 415 | Yellow-legged gull | Positive |  | 2009 | 23 | Columbretes Islands | Marine |
| 416 | Yellow-legged gull | Positive |  | 2009 | 23 | Columbretes Islands | Marine |
| 417 | Yellow-legged gull | Positive | 25 | 2010 | 23 | Delta de l'Ebre | Marine |
| 418 | Yellow-legged gull | Positive |  | 2009 | 23 | Columbretes Islands | Marine |
| 419 | Yellow-legged gull | Negative |  | 2009 | 23 | Columbretes Islands | Marine |
| 420 | Yellow-legged gull | Negative |  | 2010 | 23 | Medas Islands | Waterfresh |
| 421 | Yellow-legged gull | Negative |  | 2010 | 23 | Columbretes Islands | Marine |
| 422 | Yellow-legged gull | Negative |  | 2010 | 23 | Ons Island | Marine |
| 423 | Yellow-legged gull | Negative |  | 2010 | 23 | Columbretes Islands | Marine |
| 424 | Yellow-legged gull | Positive | 50 | 2009 | 23 | Medas Islands | Waterfresh |
| 425 | Yellow-legged gull | Positive | 50 | 2009 | 23 | Medas Islands | Waterfresh |
| 426 | Yellow-legged gull | Positive | 50 | 2009 | 23 | Medas Islands | Waterfresh |
| 427 | Yellow-legged gull | Positive | 500 | 2009 | 23 | Medas Islands | Waterfresh |
| 428 | Yellow-legged gull | Negative |  | 2010 | 24 | Medas Islands | Waterfresh |
| 429 | Yellow-legged gull | Negative |  | 2010 | 24 | Medas Islands | Waterfresh |
| 430 | Yellow-legged gull | Negative |  | 2009 | 24 | Columbretes Islands | Marine |
| 431 | Yellow-legged gull | Positive | 50 | 2009 | 24 | Delta de l'Ebre | Marine |
| 432 | Yellow-legged gull | Positive | 500 | 2009 | 24 | Delta de l'Ebre | Marine |
| 433 | Yellow-legged gull | Negative |  | 2009 | 24 | Columbretes Islands | Marine |
| 434 | Yellow-legged gull | Negative |  | 2010 | 24 | Columbretes Islands | Marine |
| 435 | Yellow-legged gull | Negative |  | 2009 | 24 | Medas Islands | Waterfresh |
| 436 | Yellow-legged gull | Negative |  | 2009 | 24 | Columbretes Islands | Marine |
| 437 | Yellow-legged gull | Negative |  | 2009 | 24 | Columbretes Islands | Marine |
| 438 | Yellow-legged gull | Positive |  | 2009 | 24 | Columbretes Islands | Marine |
| 439 | Yellow-legged gull | Negative |  | 2010 | 24 | Medas Islands | Waterfresh |
| 440 | Yellow-legged gull | Negative |  | 2009 | 24 | Columbretes Islands | Marine |
| 441 | Yellow-legged gull | Negative |  | 2009 | 24 | Delta de l'Ebre | Marine |
| 442 | Yellow-legged gull | Negative |  | 2009 | 24 | Columbretes Islands | Marine |
| 443 | Yellow-legged gull | Positive |  | 2009 | 24 | Columbretes Islands | Marine |
| 444 | Yellow-legged gull | Positive |  | 2009 | 25 | Columbretes Islands | Marine |
| 445 | Yellow-legged gull | Negative |  | 2009 | 25 | Medas Islands | Waterfresh |
| 446 | Yellow-legged gull | Negative |  | 2009 | 25 | Columbretes Islands | Marine |
| 447 | Yellow-legged gull | Positive |  | 2009 | 25 | Columbretes Islands | Marine |
| 448 | Yellow-legged gull | Negative |  | 2009 | 25 | Columbretes Islands | Marine |
| 449 | Yellow-legged gull | Negative |  | 2009 | 25 | Delta de l'Ebre | Marine |
| 450 | Yellow-legged gull | Negative |  | 2010 | 25 | Columbretes Islands | Marine |
| 451 | Yellow-legged gull | Negative |  | 2010 | 25 | Medas Islands | Waterfresh |
| 452 | Yellow-legged gull | Negative |  | 2010 | 25 | Medas Islands | Waterfresh |
| 453 | Yellow-legged gull | Negative |  | 2009 | 25 | Columbretes Islands | Marine |
| 454 | Yellow-legged gull | Negative |  | 2011 | 25 | Delta de l'Ebre | Marine |
| 455 | Yellow-legged gull | Positive | 50 | 2010 | 25 | Medas Islands | Waterfresh |
| 456 | Yellow-legged gull | Positive |  | 2009 | 25 | Columbretes Islands | Marine |
| 457 | Yellow-legged gull | Negative |  | 2010 | 25 | Columbretes Islands | Marine |
| 458 | Yellow-legged gull | Negative |  | 2010 | 25 | Columbretes Islands | Marine |
| 459 | Yellow-legged gull | Positive |  | 2009 | 26 | Columbretes Islands | Marine |
| 460 | Yellow-legged gull | Positive | 50 | 2009 | 26 | Delta de l'Ebre | Marine |
| 461 | Yellow-legged gull | Negative |  | 2010 | 26 | Columbretes Islands | Marine |
| 462 | Yellow-legged gull | Negative |  | 2009 | 26 | Columbretes Islands | Marine |
| 463 | Yellow-legged gull | Negative |  | 2009 | 26 | Columbretes Islands | Marine |
| 464 | Yellow-legged gull | Negative |  | 2009 | 26 | Columbretes Islands | Marine |
| 465 | Yellow-legged gull | Positive |  | 2009 | 26 | Columbretes Islands | Marine |
| 466 | Yellow-legged gull | Positive | 25 | 2009 | 26 | Medas Islands | Waterfresh |
| 467 | Yellow-legged gull | Negative |  | 2009 | 26 | Columbretes Islands | Marine |
| 468 | Yellow-legged gull | Positive | 100 | 2009 | 26 | Medas Islands | Waterfresh |
| 469 | Yellow-legged gull | Negative |  | 2009 | 26 | Medas Islands | Waterfresh |
| 470 | Yellow-legged gull | Negative |  | 2009 | 27 | Delta de l'Ebre | Marine |
| 471 | Yellow-legged gull | Positive |  | 2009 | 28 | Columbretes Islands | Marine |
| 472 | Yellow-legged gull | Positive |  | 2009 | 28 | Columbretes Islands | Marine |
| 473 | Yellow-legged gull | Positive | 25 | 2009 | 28 | Delta de l'Ebre | Marine |
| 474 | Yellow-legged gull | Negative |  | 2010 | 28 | Columbretes Islands | Marine |
| 475 | Yellow-legged gull | Positive |  | 2009 | 29 | Columbretes Islands | Marine |
| 476 | Yellow-legged gull | Negative |  | 2009 | 29 | Medas Islands | Waterfresh |
| 477 | Yellow-legged gull | Negative |  | 2010 | 29 | Medas Islands | Waterfresh |
| 478 | Yellow-legged gull | Negative |  | 2010 | 29 | Medas Islands | Waterfresh |
| 479 | Yellow-legged gull | Negative |  | 2009 | 30 | Columbretes Islands | Marine |
